# Role of *RASEF* hypermethylation in cigarette smoke-induced pulmonary arterial smooth muscle remodeling

**DOI:** 10.1186/s12931-019-1014-1

**Published:** 2019-03-07

**Authors:** Qinghai Li, Jixing Wu, Yongjian Xu, Lu Liu, Jungang Xie

**Affiliations:** 1 Department of Respiratory and Critical Care Medicine, Tongji Hospital, Tongji Medical College, Huazhong University of Science and Technology, Wuhan, 430030 China; 2 Department of Pulmonary Medicine, Qingdao Municipal Hospital, School of Medicine, Qingdao University, Qingdao, 266011 China; 3 Department of Pharmacy, Tongji Hospital, Tongji Medical College, Huazhong University of Science and Technology, Wuhan, 430030 China

**Keywords:** Pulmonary hypertension, Cigarette smoke, Pulmonary arterial smooth muscle, DNMT1, RASEF

## Abstract

**Background:**

Pulmonary hypertension (PH) is a progressive and fatal disease. While cigarette smoke can change DNA methylation status, the role of such molecular alterations in smoke-associated PH is unclear.

**Methods:**

A PH rat model was developed by exposing animals to cigarette smoke for 3 months. Right ventricular systolic pressure was measured with a right heart catheter. Histological changes (right ventricular hypertrophy index, medial wall thickness in pulmonary arteries (PAs)) and DNMT1 protein levels in rat PAs or primary human PA smooth muscle cells (HPASMCs) exposed to cigarette smoke extract were assessed. Methylation sequencing and MassArray® were used to detect genomic and *RASEF* promoter methylation status, respectively. After DNMT1 knockdown and cigarette smoke extract exposure, HPASMCs behavior (proliferation, migration) and *RASEF* methylation status were examined; RASEF mRNA expression was evaluated by real-time-polymerase chain reaction. RASEF overexpression viral vectors were used to assess the impact of RASEF on rat PH and HPASMCs remodeling.

**Results:**

Higher right ventricular systolic pressure, medial wall thickness, and right ventricular hypertrophy index values were observed in the smoking group rats. Smoke exposure increased DNMT1 expression and *RASEF* methylation levels in rat PAs and HPASMCs. Cigarette smoke extract induced HPASMCs behavioral changes and *RASEF* hypermethylation followed by silencing, while DNMT1 knockdown markedly inhibited these changes. RASEF overexpression distinctly inhibited PH and HPASMCs remodeling, possibly through phospho-AKT (Ser473), PCNA, and MMP9 downregulation.

**Conclusions:**

Cigarette smoke caused PA remodeling in PH rats related to *RASEF* hypermethylation. These results expand our understanding of key epigenetic mechanisms in cigarette smoke-associated PH and potentially provide a novel therapeutic target for PH.

**Electronic supplementary material:**

The online version of this article (10.1186/s12931-019-1014-1) contains supplementary material, which is available to authorized users.

## Introduction

Pulmonary hypertension (PH) is defined as an increase in mean pulmonary arterial pressure above 25 mmHg at rest. PH is a complex, deadly, and refractory disease associated with various respiratory and cardiovascular diseases [1]. An estimated 30–50% of patients with advanced chronic obstructive pulmonary disease (COPD) suffer from PH [2, 3], while 1–3% patients with mild COPD have severe PH [4]. Few studies have investigated the molecular mechanisms of PH associated with COPD (PH-COPD).

Pulmonary arterial smooth muscle remodeling (PASMR) can lead to media thickening, a main pathological characteristic of PH [5, 6], due to increased proliferation, apoptotic resistance, and migration of PA smooth muscle cells (SMCs) [7]. Except for hypoxia [8], cigarette smoking (the major risk factor for COPD) can directly facilitate the remodeling of PA smooth muscle (PASM) [9, 10] and could partly explain the pathogenesis of PH-COPD.

DNA methylation, the most common genome epigenetic modification that can regulate chromosomal stability and gene expression under the catalytic action of DNA methyltransferases (DNMTs) [11]; eukaryotic DNMTs consist of a maintenance methyltransferase (DNMT1) and two de novo methyltransferases (DNMT3a and DNMT3b) [12]. Genomic methylation status can be easily altered by environmental factors [13]. Cigarette and tobacco smoke have been shown to strongly modify genome methylation [14, 15], which is related to many diseases, such as COPD [16], lung cancer [17], and atherosclerosis [18]. Altered DNA methylation (hypermethylation) has also been shown to play an important role in group I PH [19, 20]. DNMT1 overexpression has been found in PH tissues and involved in hypoxia-induced HPASMCs proliferation and apoptotic resistance [21]. 5-Azacytidine (5-Aza), a selective DNMT1 inhibitor, has been shown to inhibit platelet-derived growth factor-induced aortic and airway SMC proliferation and migration, and has a protective role in atherosclerosis and asthma [22, 23]. However, the roles of DNMT1 in cigarette smoke (CS)-associated PH have not been investigated.

Rab GTPases are vital regulators of intracellular membrane traffic [24], and Rab proteins influence the transport and/or function of signal transducers and growth factors. RASEF (also known as Rab45) is an atypical GTPase, as a tumor suppressor gene in cutaneous malignant melanoma [25]. Maat et al. found *RASEF* promotor hypermethylation was inversely related with the survival of uveal melanoma patients [26]. In addition, RASEF was shown to distinctly promote apoptosis of chronic myeloid leukemia progenitor cells via activation of p38 signal [27].

As a proliferative disease similar to tumors, PH may also be improved by many tumor suppressor genes, such as P53, P21 and PPAR-γ [28, 29]. However, the relationship between RASEF and PH is unknown. Therefore, this study investigated the role of RASEF on CS-induced remodeling of PASM and rat PH.

## Materials and methods

### Animals models

Adult male Sprague–Dawley rats (180–220 g) were acquired from the Experimental Animal Center of Tongji Medical College (Wuhan, China). All animal experiments were carried out according to the Animal Care and Use guidelines of the Chinese Council on Animal Care. Twenty rats were randomly and equally divided into two groups (*n* = 10 each) and exposed to either fresh air (the air group) or cigarette smoke (the smoking group). The smoking group rats were exposed to the smoke from 10 cigarettes (Hong Jin Long, 1.2 mg nicotine, 15 mg tar per cigarette, Wuhan, China) in a ventilated whole body exposure chamber for 1 h each time, two times per day for a total of 3 months according to previous protocols [30, 31]. All rats were euthanized with sodium pentobarbital.

### RASEF overexpression in PH rats

Thirty rats were randomly divided into two groups and exposed to fresh air (*n* = 10) or cigarette smoke (*n* = 20) as described above. Then, rats exposed to cigarette smoke were equally subdivided (*n* = 10 each) and infected with either adeno-associated virus type-1 (Hanheng Biotechnology, China) expressing green fluorescent protein (AAV1.GFP) or AAV1 containing rat *RASEF* cDNA (AAV1.RASEF) by tracheal injection (1 × 10^11^ viral genomes/rat) as described previously [32]; the air group rats received AAV1.GFP. All rats were sacrificed by sodium pentobarbital 6 weeks after infection.

### Hemodynamic measurements and histological analysis

A 3F polyethylene catheter and the PowerLab system (AD Instruments, Australia) were used to test right ventricular (RV) systolic pressure (RVSP) in vivo as described previously [33]. After hemodynamic measurements were completed, rats were sacrificed as described above and hearts divided into the RV and left ventricle plus septum (LV + S). RV and LV + S tissues weighed and used to calculate the RV hypertrophy index (RVHI), which is the mass ratio of the RV to the LV + S. Left lung tissue was fixed, and 4-μm paraffin sections were made and stained with hematoxylin and eosin. The wall thickness of pulmonary arterioles (outside diameter: 50–150 μm) was then measured using an optical microscope (Olympus BX61, Tokyo, Japan) [34].

### HPASMCs culture and transfection

HPASMCs were purchased from American Type Culture Collection (MD, USA) and grown in Dulbecco’s Modified Eagle’s Medium-F12 containing 10% fetal bovine serum. Cigarette smoke extract (CSE) obtained from Research Cigarettes (Code 3R4F, University of Kentucky, USA) was acquired as described elsewhere [35]. DNMT1 small interfering RNA (siRNA; 50 nM) was transfected into HPASMCs using Lipofectamine 2000 (Invitrogen, USA) for silencing DNMT1. DNMT1 siRNA target sequences were as follows: the first 5′-GCACCUCAUUUGCCGAAUATT-3′; the second 5′- GGGACUGUGUCUCUGUUAUTT-3′. DNMT1 overexpression pcDNA3.1 plasmid vector was also transfected into HPASMCs using Lipofectamine 2000 (Vigene Biosciences, China). The RASEF overexpression adenovirus vector (Ad.RASEF; Vigene Biosciences, China) was also transfected into HPASMCs (MOI 250).

### Western blot

Total proteins were extracted from rat PAs or HPASMCs, and their concentrations were measured with a BCA kit (Servicebio, China). Primary antibodies against β-actin (Sungene, China), DNMT1 (ABclonal, USA), RASEF, matrix metalloproteinase 9 (MMP9) (Abcam, UK), phospho-AKT (ser-473), AKT (Cell Signaling Technologies, USA), and proliferating cell nuclear antigen (PCNA; Proteintech, China) were used. Bands were detected by a ChemiDoc MP System (Bio-Rad Laboratories, USA), and the intensity was analyzed by ImageJ software.

### Real-time polymerase chain reaction (RT-PCR)

Total RNA was extracted from rat PAs and HPASMCs, reverse transcribed, and then subjected to RT-PCR using corresponding kits according to the manufacturer’s instructions (Takara, Japan). The primers used were as follows: human β-actin, 5′-AGAAAATCTGGCACCACACCT-3′ (forward) and 5′-GATAGCACAGCCTGGATAGCA-3′ (reverse); rat β-actin, 5′-CGTAAAGACCTCTATGCCAACA-3′ (forward) and 5′-CGGACTCATCGTACTCCTGCT-3′ (reverse); human *DNMT1*, 5′-AGGCGGCTCAAAGATTTGGAA-3′ (forward) and 5′-GCAGAAATTCGTGCAAGAGATTC-3′ (reverse); human *RASEF*, 5′-AGATTGTACTTGCTGGGGACG-3′ (forward) and 5′-GAGCTGCAGAACTGTTCGTT-3′ (reverse); rat *RASEF*, 5′-ACGGGATCTGGAACTAATCCG-3′ (forward) and 5′-GGCACTTCTAAGGCCGTCAT-3′ (reverse). The ratio for the mRNA of interest was normalized by β-actin.

### Immunofluorescence and immunohistochemistry

Rat lung sections were stained with mouse anti-α-smooth muscle actin (Abcam, UK) and rabbit anti-RASEF (Abcam, UK) primary antibodies followed by fluorescein isothiocyanate-conjugated anti-mouse immunoglobulin G and phycoerythrin-conjugated anti-rabbit immunoglobulin G (Jackson ImmunoResearch, USA). HPASMCs were incubated with a primary anti-DNMT1 (ABclonal, USA) antibody followed by phycoerythrin-conjugated anti-rabbit immunoglobulin G. And 4′,6-diamidino-2-phenylindole was used to stain nuclei. Immunofluorescence was observed by fluorescence microscope. Immunohistochemical staining was performed according to the manufacturer’s instructions (Boster Biological Technology, China). Primary antibodies against RASEF, MMP9 (Abcam, UK), and phospho-AKT (ser-473) (Cell Signaling Technology, USA) were used.

### DNA methylation analysis

Genomic DNA of rat PAs was extracted using a QIAamp® DNA Mini kit (Qiagen, Germany) according to the manufacturer’s instructions, and genome-wide methylation sequencing using MethylRAD technology was carried out by the Shanghai OE Biotech Company (China). The CpG methylation status of *RASEF* promotor (from − 224 to + 196 bp) in rat PAs was determined by a MassARRAY® system, which was carried out by the OE Biotech Company. The methylated primers used were as follows: forward 5′- aggaagagag GTATTTGGGAATGAGTTGGGTT-3′, and reverse 5′- cagtaatacgactcactatagggagaaggct CAACAACAATTAAAAACAAAAACCTC-3′. HPASMCs were pretreated with 2 μM 5-Aza or DNMT1 siRNA for 24 h and then incubated with 2% cigarette smoke extract (CSE) for 24 h. Cellular DNA was extracted as described above, and the CpG methylation status of the *RASEF* promotor (from − 164 to + 215 bp) was detected by Compass Biotechnology (Beijing, China) using a MassARRAY® system. The methylated primers used were 5′-aggaagagagGTAGGGTTTTTTTTGGAAGGA-3′ (forward) and 5′-cagtaatacgactcactatagggagaaggctCAACCAAATCTCCCCCACCTAC-3′ (reverse).

### Cellular viability and cell cycle assay

In order to study the role of DNMT1 on HPASMCs proliferation, HPASMCs were treated with 2 μM 5-Aza or DNMT1 siRNA for 24 h followed by incubation with 2% CSE for 48 h. Cell proliferation/viability was then assessed with a Cell Counting Kit-8 (CCK8; Promotor, China) and cell cycle detection kit (KeyGEN BioTECH, China) according to the manufacturer’s instructions. CCK8 and cell cycle analyses were completed using an automated spectrophotometric plate reader (PerkinElmer, USA) and flow cytometer (BD Bioscience, USA), respectively. In addition, the proliferation of HPASMCs infected with Ad.NC or Ad.RASEF for 24 h then 2% CSE for another 48 h was measured by cell cycle analysis and 5-ethynyl-2′-deoxyuridine (EdU) staining (RiboBio, China). EdU was labeled with Apollo 567, and cells were observed via fluorescence microscope.

### Cellular apoptosis detection

HPASMCs were transfected with Ad.RASEF for 24 h followed by 2% CSE for another 48 h. Then, cells were harvested, and annexin V-fluorescein isothiocyanate and propidium iodide were added to the cell suspension. Flow cytometry was used to measure apoptosis. Experiments were repeated at least three times.

### Migration assay

Membranes with 8-μm pores in 24-well Transwell® plates (Coring, USA) were employed for migration assays. HPASMCs were transfected with DNMT1 siRNA or Ad.RASEF for 24 h before being digested and counted. About 50,000 cells were added to the upper chamber for 24 h to adhere. Next, 600 μL of standard culture medium with or without 2% CSE was added to the lower compartment, and the upper chamber liquid was replaced with 200 μL of medium including 5% fetal bovine serum. After 24 h, cells within the membranes of bottom chambers were fixed, stained with 0.1% crystal violet, and imaged. Experiments were repeated at least three times.

### Statistical analysis

Data were analyzed using GraphPad Prism software (version 5.0) and expressed as the mean ± standard error of mean. Student’s *t*-test was used for comparisons between two groups. One-way analysis of variance followed by a Newman–Keuls post-test was utilized for multiple group comparisons. A *P* < 0.05 was considered statistically significant.

## Results

### Cigarette smoke induced PH in rats

**Fig. 1:**
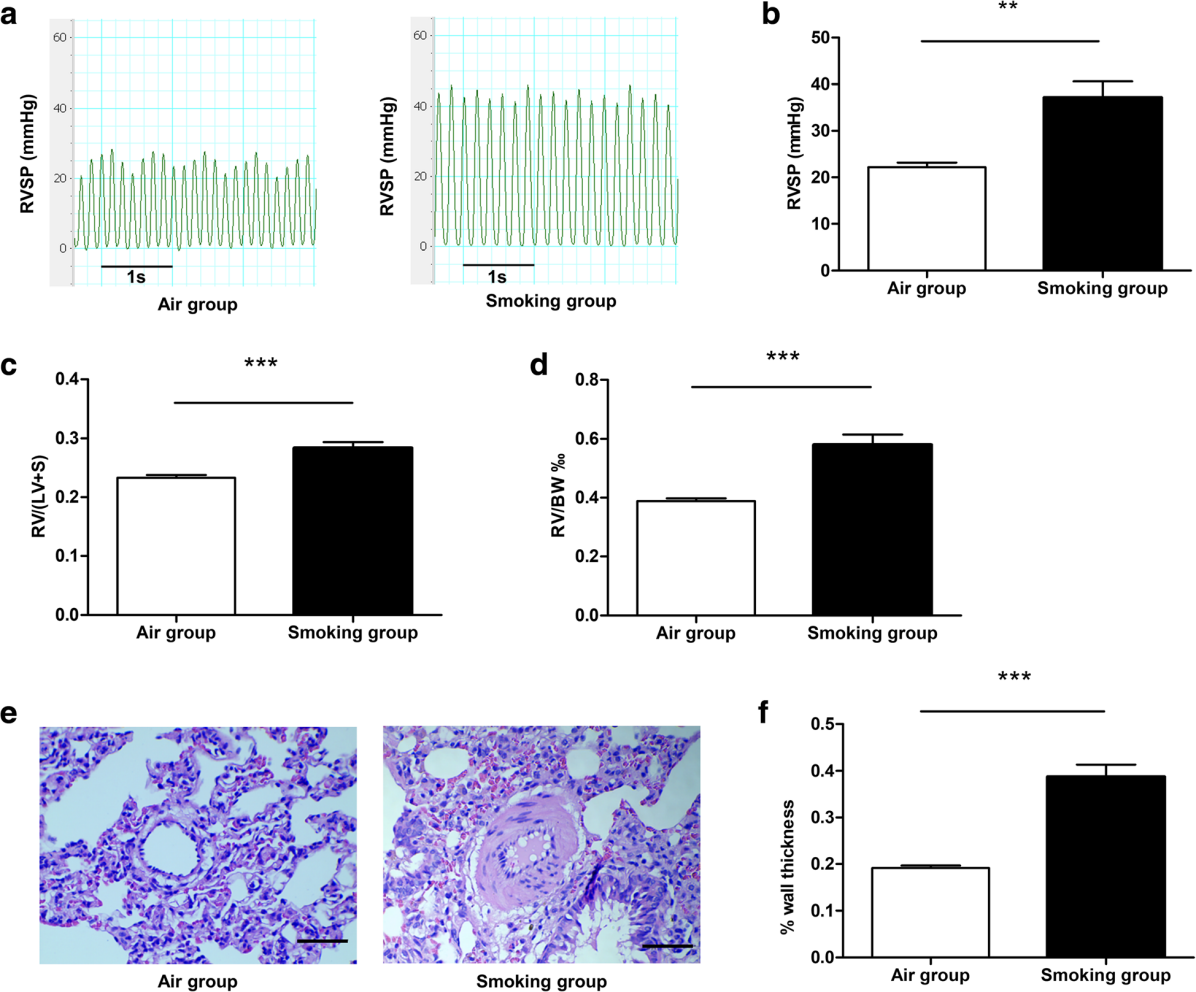
Influence of cigarette smoke on rat hemodynamics and histopathology. Rat RVSP (**a**, **b**), RVHI [RV to LV + S mass ratio] (**c**), RV to body weight mass ratio (**d**), and pulmonary arteriole wall thickness (**e**, **f**). Scale bar = 50 μm. Data are displayed as the mean ± standard error of the mean (*n* = 10 per group); **P < 0.01, ***P < 0.001 versus control

The RVSP was significantly higher in the smoking group (37.2 ± 3.5 mmHg) than in the air group (22.2 ± 1.0 mmHg, *P* < 0.01, Fig. 1a, b). The RVHI and mass ratio of the RV to body weight were increased in the smoking group (Fig. 1c, d). The wall thickness of pulmonary arterioles was also obviously increased in the smoking group (Fig. 1e, f).

### Cigarette smoke increased DNMT1 expression in pulmonary arterial smooth muscle

**Fig. 2:**
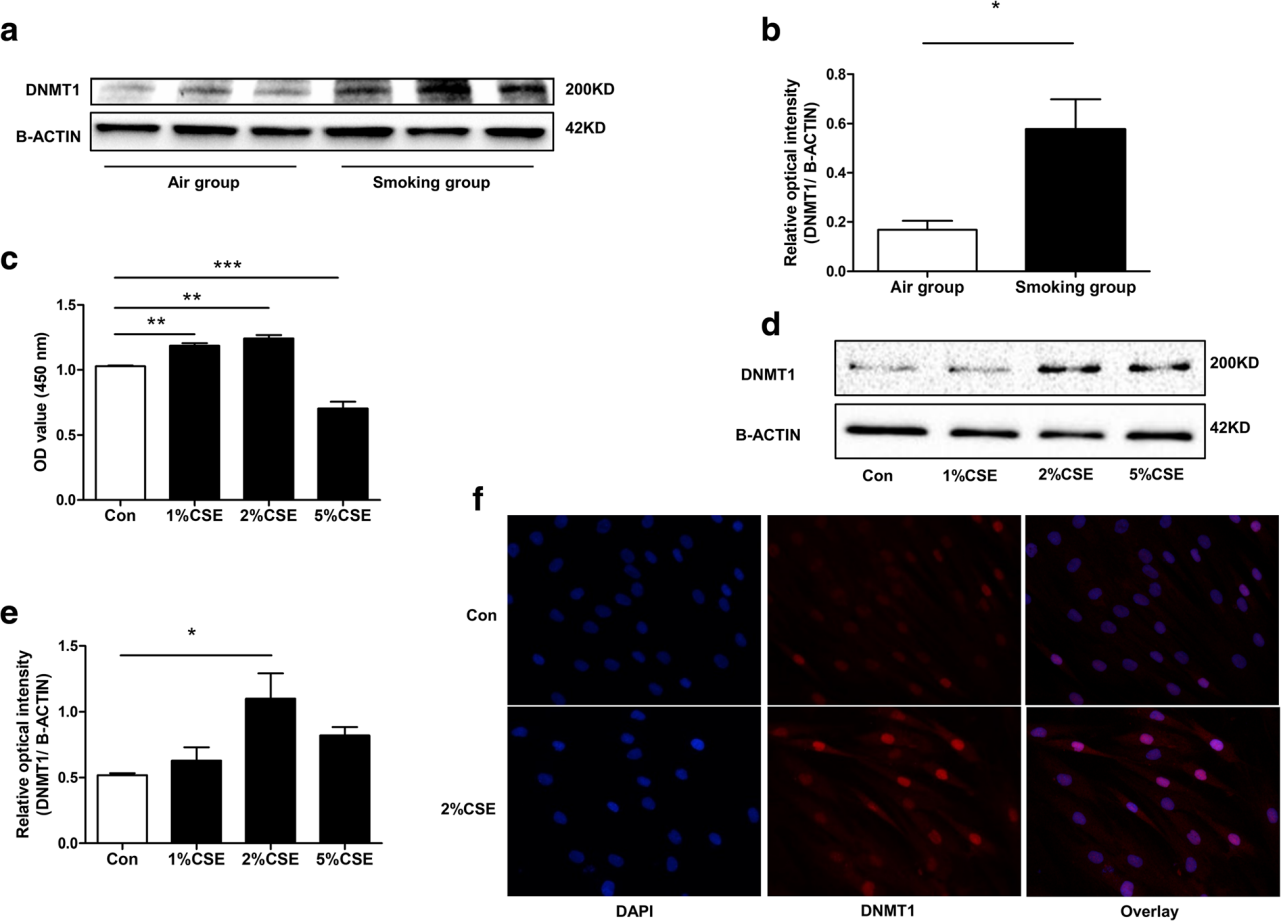
Effect of cigarette smoke on DNMT1 levels in rat PAs and HPASMCs. **a**, **b** Western blot showed that cigarette smoke increased DNMT1 protein levels in rat PAs. **c** CCK8 assay revealed an increase in HPASMCs cell viability after treatment with different concentrations of CSE for 48 h. **d**, **e** DNMT1 protein expression in HPASMCs treated with CSE for 48 h. **f** Immunofluorescence results indicate an increase in DNMT1 protein levels (increased fluorescence) in CSE-treated HPASMCs. Data are displayed as the mean ± standard error of the mean (*n* = 3); (**b**) *P < 0.05 vs. the air group; (**c**, **e**) *P < 0.05, **P < 0.01, ***P < 0.001 vs. Con

DNMT1 protein were higher in rats PA smooth muscle of the smoking group than controls (Fig. 2a, b). In addition, exposure of HPASMCs to 2%CSE increased cell viability (Fig. 2c) and DNMT1 protein levels (Fig. 2d-f).

### 5-Aza and DNMT1 siRNA inhibited CSE-induced HPASMCs viability, cell cycle, migration

**Fig. 3:**
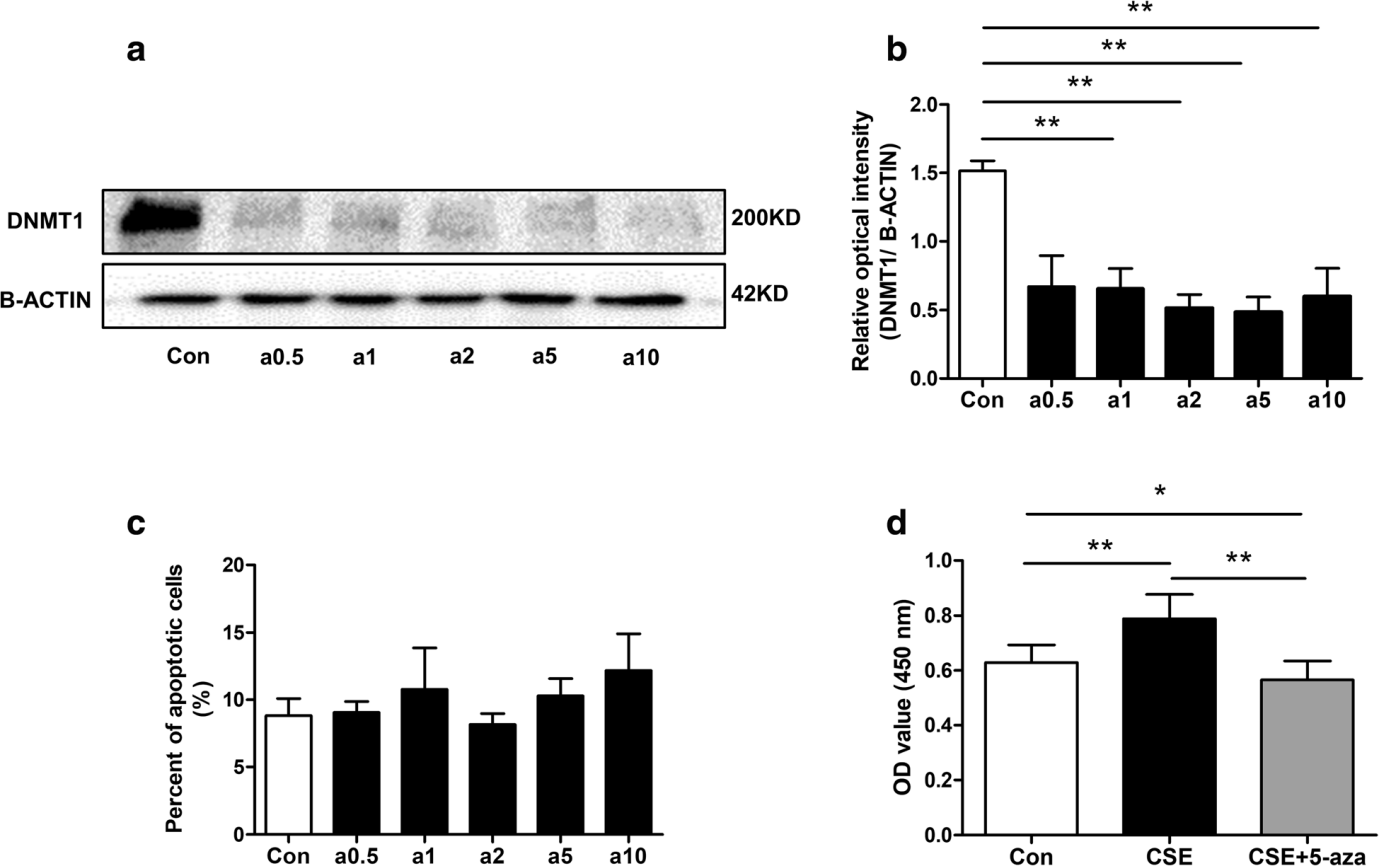
Effect of 5-Aza on CSE-induced HPASMCs proliferation and apoptosis changes. HPASMCs pretreatment with various concentration of 5-Aza (0, 0.5, 1, 2, 5, and 10 μM) for 48 h decreased DNMT1 protein levels (**a**, **b**) but did not affect the number of apoptotic cells (**c**). **d** CCK8 assay revealed pretreatment with 5-Aza (2 μM) inhibited CSE-induced increases in HPASMCs viability. Data are displayed as the mean ± standard error of the mean (*n* = 3); *P < 0.05, **P < 0.01

**Fig. 4:**
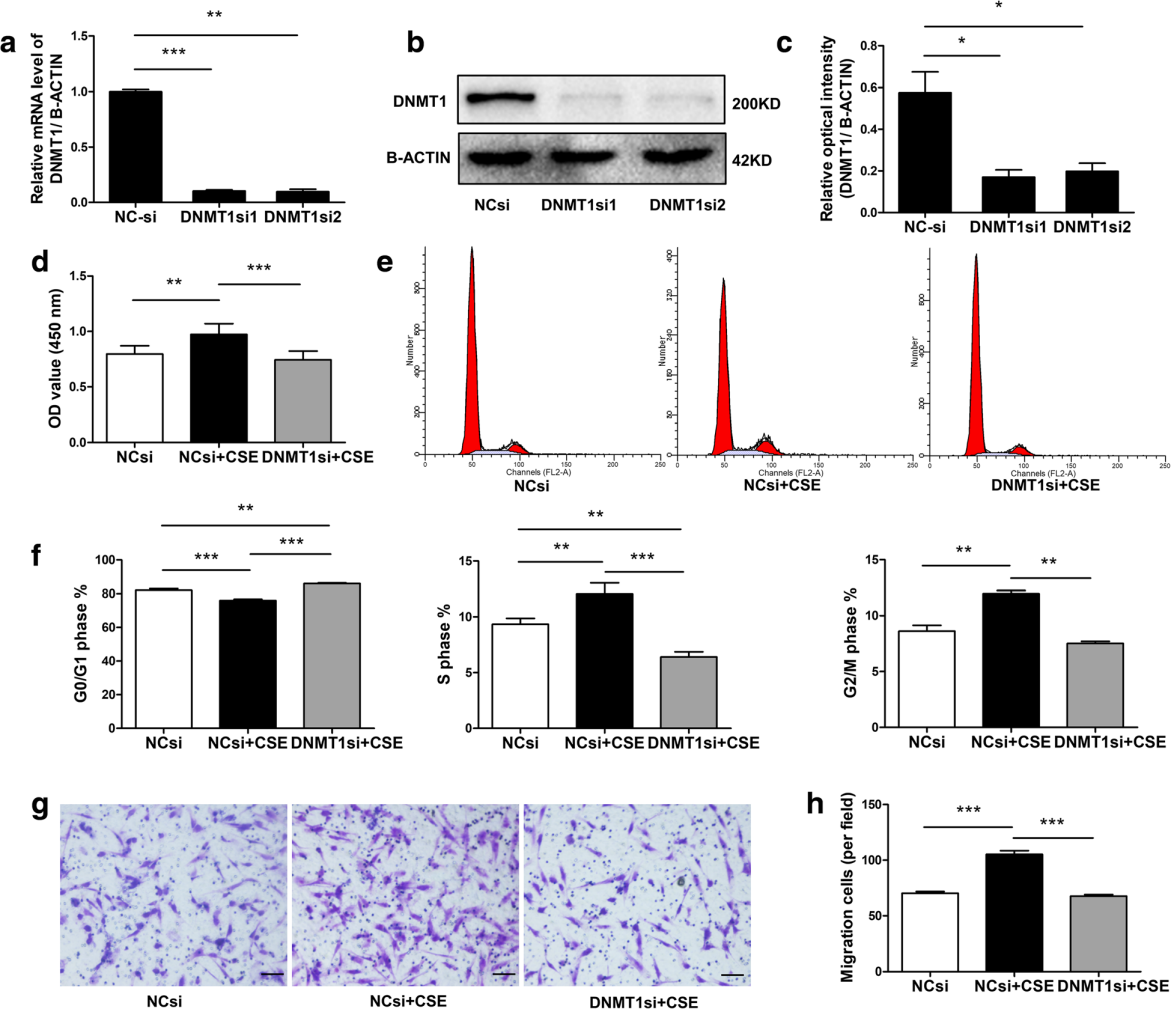
Effect of DNMT1 siRNA on CSE-induced HPASMCs proliferation and migration changes. **a**-**c** DNMT1 siRNA reduced DNMT1 mRNA and protein expression in HPASMCs. Pretreatment of HPASMCs with DNMT1 siRNA suppressed 48-h CSE-induced increases in cell viability (**d**) and cell cycle transition from G0/G1 to S or G2/M phase (**e**, **f**). **g**, **h** DNMT1 siRNA transfection prevented 24-h CSE-induced migration of HPASMCs. Scale bar = 100 μm. Data are displayed as the mean ± standard error of the mean (*n* = 3); *P < 0.05, **P < 0.01, ***P < 0.001

5-Aza (0.5–10 μM), a selective inhibitor of DNMT1, and DNMT1 siRNA both reduced DNMT1 protein levels in HPASMCs (Figs. 3a, b and 4a-c, respectively). Pretreatment with 5-Aza (2 μM) inhibited CSE-induced the increase in cell viability (Fig. 3d) but did not influence apoptosis (Fig. 3c) in HPASMCs. DNMT1 siRNA also suppressed CSE-induced proliferation and cell cycle transition from G0/G1 to S or G2/M phase (Fig. 4d-f). DNMT1 siRNA also inhibited CSE-induced cell migration (Fig. 4g, h).

### Cigarette smoke reduced RASEF expression in rat PAs

**Fig. 5:**
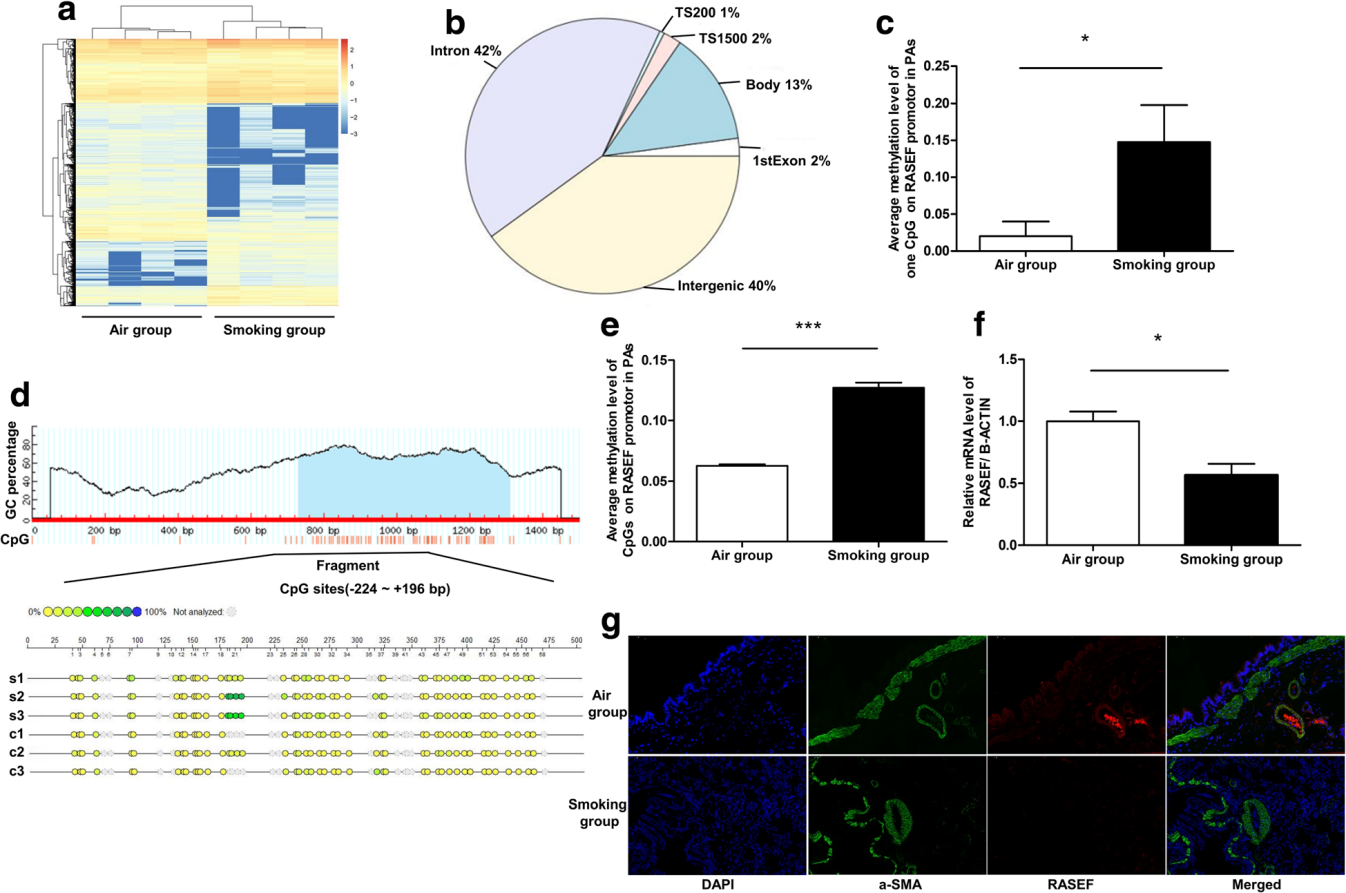
Cigarette smoke influenced *RASEF* promoter methylation and transcription in rat PAs. Genome-wide DNA methylation sequencing and heat map of rat PAs revealed a number of changes in gene distribution associated with different methylation sites (**a**, **b**). **c** Cigarette smoke caused hypermethylation of a CpG within the *RASEF* promoter (located at NC_005104.4–90,418,763) in rat PAs. **d**, **e** The methylation status of CpGs in *RASEF* promotor in rat PAs was measured by MassARRAY®. **f** RASEF mRNA levels were lower in the smoking PH versus control rat PAs. **g** RASEF immunofluorescence was much lower in the pulmonary arterioles of PH rats versus controls. Data are displayed as the mean ± standard error of the mean (*n* = 4); *P < 0.05, ***P < 0.001 vs. Air group

DNA methylation sequencing based on MethylRAD technology revealed many differences in genes and/or promotors (TSS200 and TSS1500) in rat PAs of the air group and the smoking group (Fig. 5a-b). And the detailed data was in Additional file 1. Among the different loci, one CpG of the *RASEF* promotor (Rnor_6.0: chromosome 5, NC_005104.4–90,418,747) was found to be hypermethylated in PAs of the smoking group (Fig. 5c). *RASEF* promoter in rat PAs of the smoking group was hypermethylated (Fig. 5d, e). Moreover, RASEF mRNA level in rat PAs in the smoking group was lower (Fig. 5f). RASEF-stained smooth muscle cells in PAs in the smoking group were also decreased (Fig. 5g).

### DNMT1 knockdown inhibited CSE-induced RASEF downregulation in HPASMCs

**Fig. 6:**
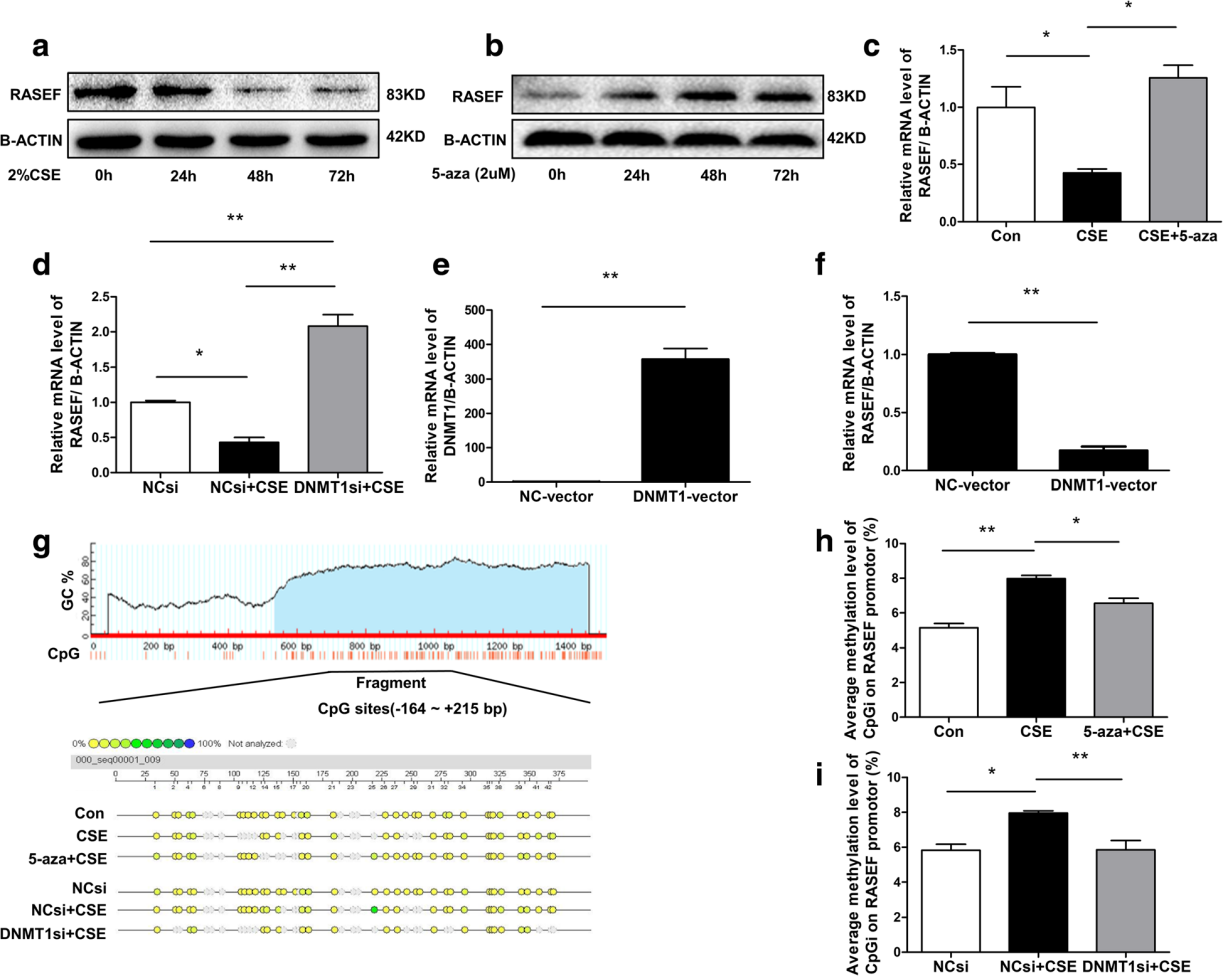
Influence of DNMT1 siRNA and 5-Aza on CSE-induced *RASEF* hypermethylation and transcription in HPASMCs. CSE reduced RASEF protein expression in HPASMCs (**a**), while 5-Aza (2 μM) increased them (**b**). **c**, **d** Pretreatment with either 5-Aza (2 μM) or DNMT1 siRNA inhibited CSE-induced downregulation of RASEF mRNA. **e**, **f** HPASMCs transfected with a DNMT1 overexpression vector reduced RASEF mRNA expression. **g**, **i** CSE-induced *RASEF* promoter CpG hypermethylation was prevented by pretreatment with either DNMT1 siRNA or 5-Aza (2 μM). Data are displayed as the mean ± standard error of the mean (*n* = 3); *P < 0.05, **P < 0.01

RASEF protein level in HPASMCs was reduced after stimulation with 2% CSE for 48 h or 72 h (Fig. 6a). Similarly, DNMT1 overexpression via transfection of DNMT1-vector significantly reduced RASEF mRNA expression (Fig. 6e, f). Treatment with 5-aza (2 μM) for 24–72 h increased RASEF protein expression (Fig. 6b). 2% CSE also inhibited RASEF mRNA expression in HPASMCs; however, 5-Aza or DNMT1siRNA could partly reverse this effect (Fig. 6c, d). More importantly, the CpG island in the *RASEF* promotor (from − 164 to + 215 bp) was hypermethylated under CSE treatment, while pretreatment of HPASMCs with either DNMT1 siRNA or 5-Aza suppressed the methylation caused by CSE (Fig. 6g-i).

### AAV1.RASEF inhibited cigarette smoke-induced PH pathology

**Fig. 7:**
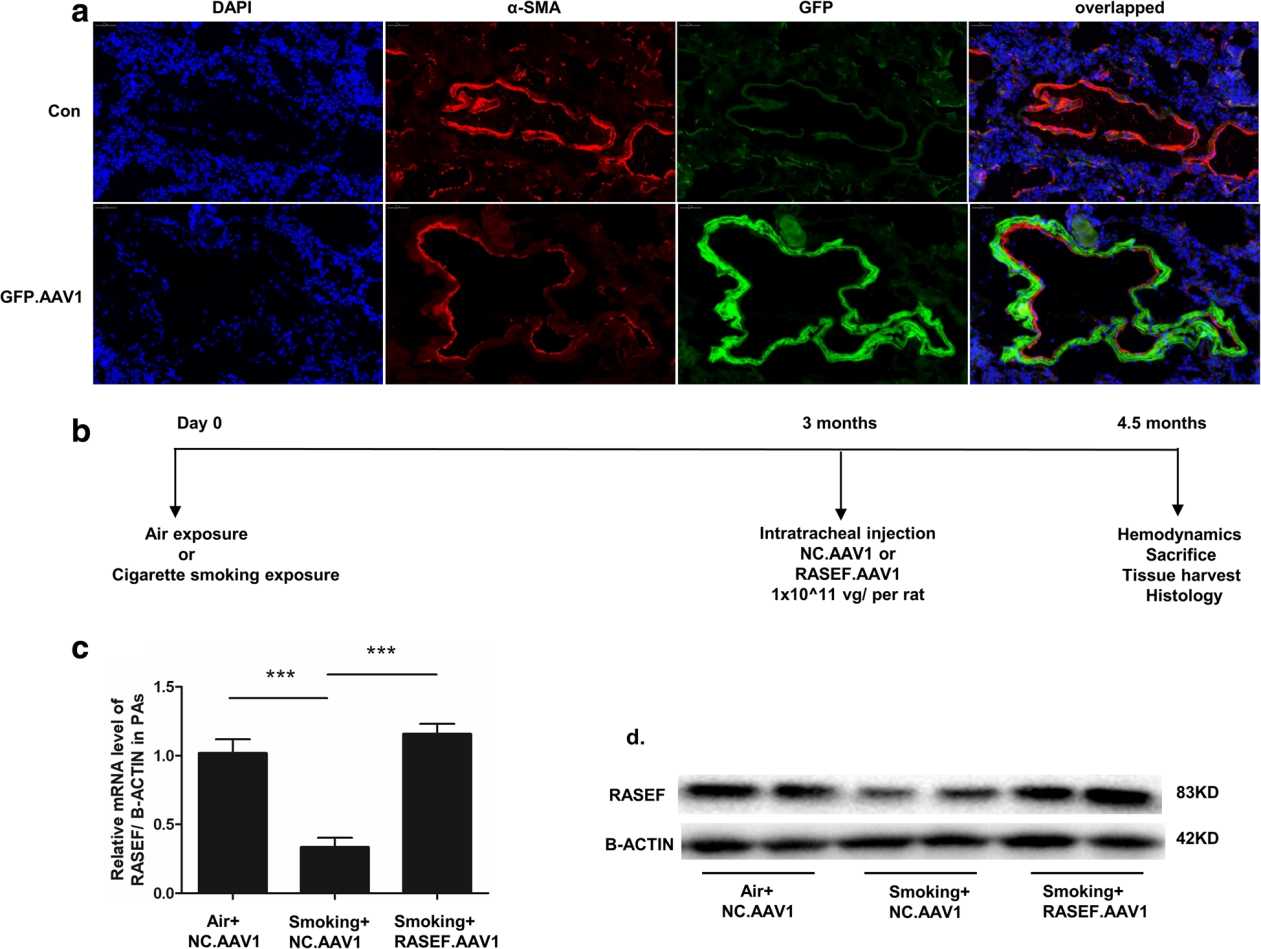
Effect of AAV1.RASEF on cigarette smoke-induced RASEF downregulation. **a** One month after tracheal injection of AAV1, the rat PAs were successfully infected. **b** Intervention plan for experimental rats. **c** AAV1.RASEF inhibited cigarette smoke-induced RASEF gene silencing in rat PAs. **d** The protein expression of RASEF in rat PAs. Data are displayed as the mean ± standard error of the mean(*n* = 6); ***P < 0.001

**Fig. 8:**
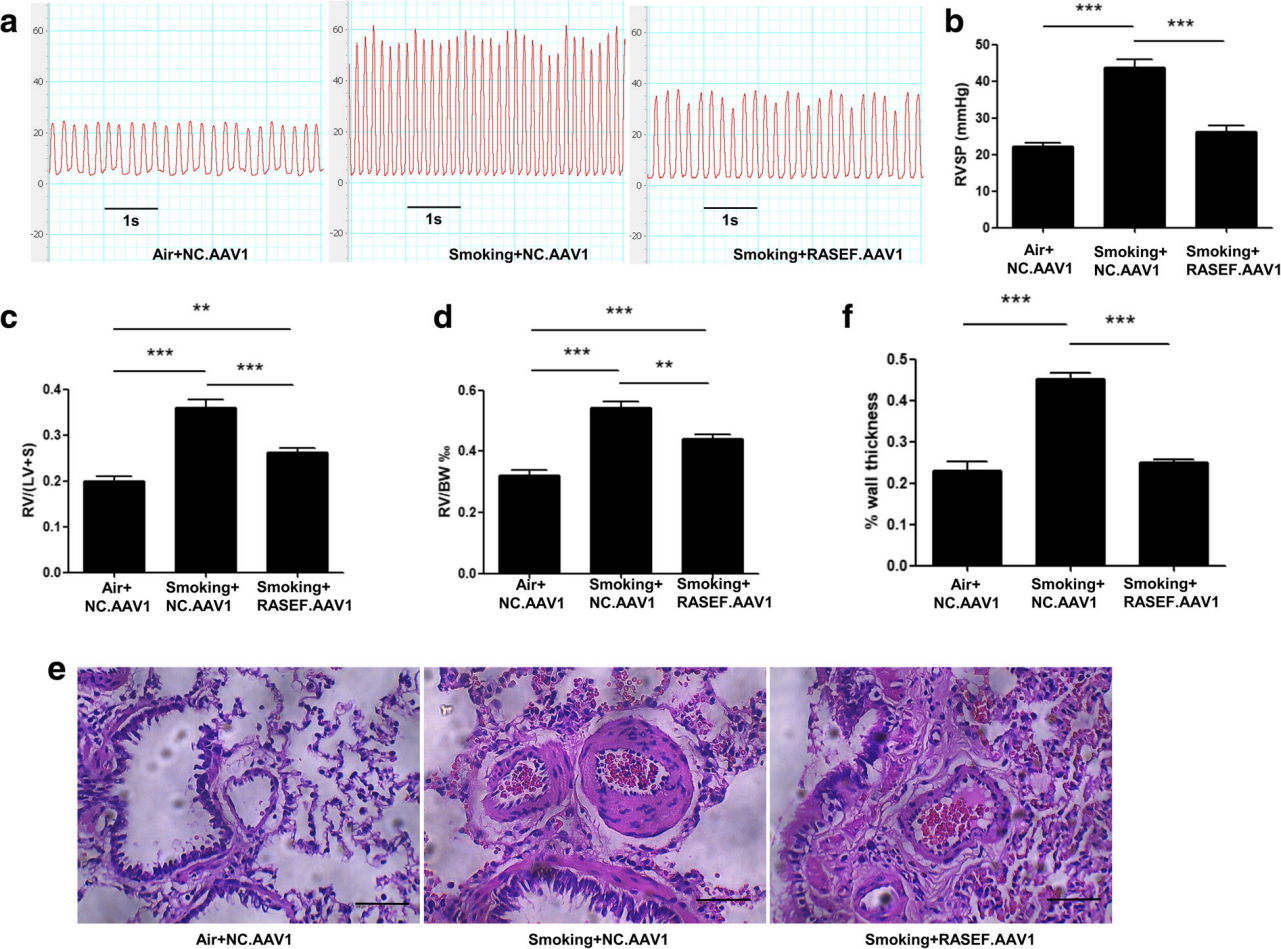
AAV1.RASEF effect on cigarette smoke-triggered rat PH. AAV1.RASEF infection significantly improved cigarette smoke-induced changes in the RVSP (**a**, **b**), RVHI [RV to LV + S mass ratio] (**c**), RV to body weight mass ratio (**d**), and wall thickness of rat pulmonary arterioles (**e**, **f**). Scale bar = 50 μm. Data are displayed as the mean ± standard error of the mean (*n* = 8); **P < 0.01, ***P < 0.001

Tracheal injection of AAV1 enabled successful infection of rat PA smooth muscle (Fig. 7a). Infection with AAV1.RASEF was found to reverse cigarette smoke (CS)-induced downregulation of RASEF (Fig. 7c-d). Moreover, both RVSP changes and RV hypertrophy caused by CS were significantly improved in AAV1.RASEF-treated rats (Fig. 8a-d). AAV1.RASEF infection also obviously reduced the thickness of pulmonary arteriole walls (Fig. 8e, f).

### Ad.RASEF inhibited CSE-induced HPASMCs cell cycle and migration changes

**Fig. 9:**
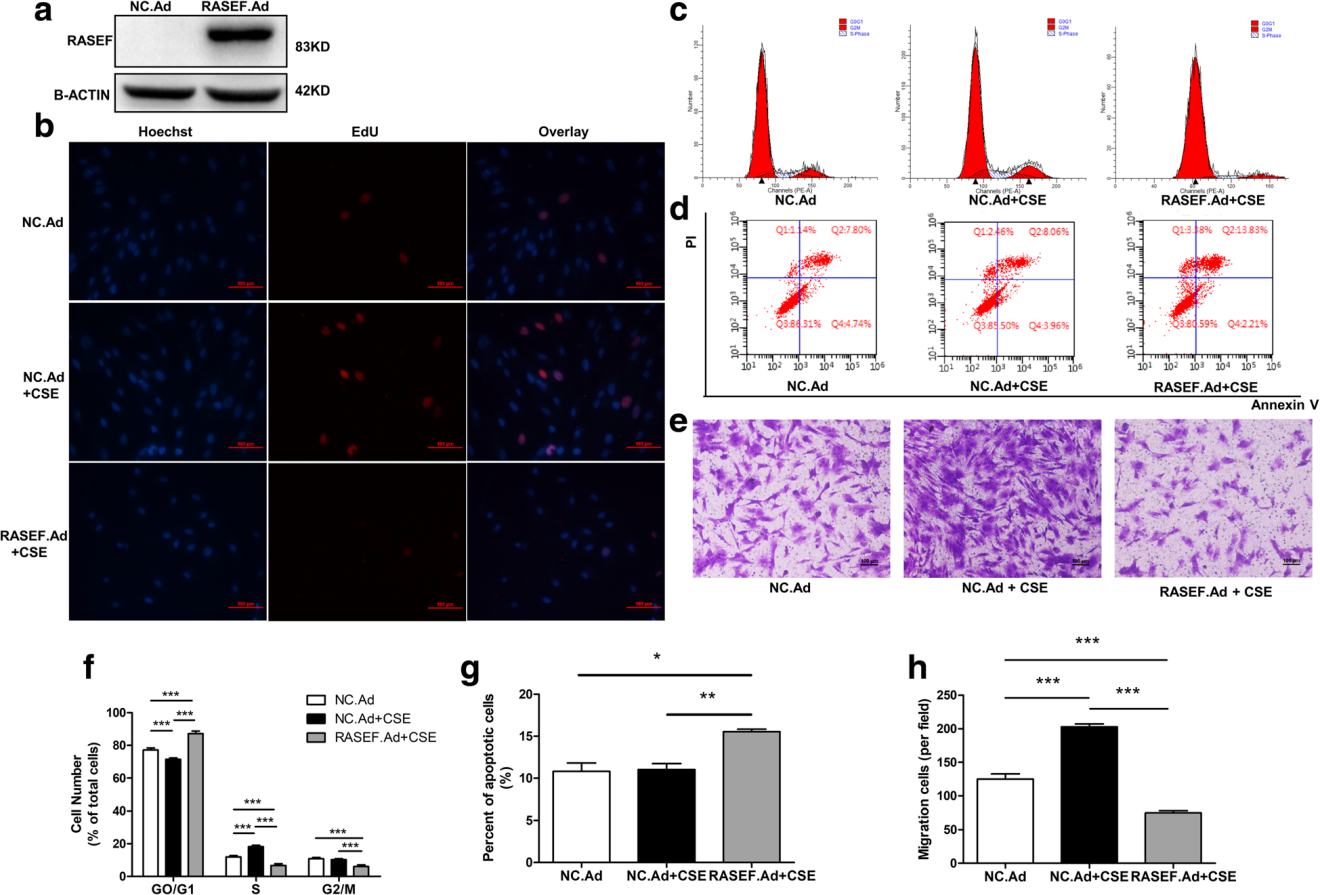
Influence of Ad.RASEF on CSE-induced HPASMCs proliferation and migration changes. **a** Ad.RASEF transfection increased RASEF protein levels in HPASMCs. Transfection of HPASMCs with Ad.RASEF inhibited CSE-induced increases in cell proliferation [EdU positivity] (**b**), reduced S and G2/M phase and increased G0/G1 phase populations (**c**, **f**), promoted apoptosis (**d**, **g**), and prevented HPASMCs migration (**e**, **h**). Scale bar = 100 μm. Data are displayed as the mean ± standard error of the mean (*n* = 3); *P < 0.05, **P < .001, ***P < 0.001

Ad.RASEF transfection successfully induced overexpression of RASEF protein in HPASMCs (Fig. 9a). Increase in EdU-positive staining cells caused by CSE treatment was significantly inhibited by Ad.RASEF transfection (Fig. 9b). CSE-induced cell cycle changes, including increased S phase and reduced G0/G1 phase populations, were also reversed by Ad.RASEF; and Ad.RASEF also reduced G2/M phase populations (Fig. 9c, f). Moreover, Ad.RASEF promoted HPASMCs apoptosis (Fig. 7d, g), and CSE-induced HPASMCs migration was reversed by Ad.RASEF (Fig. 9e, h).

### RASEF overexpression reduced cigarette smoke -induced AKT activity

**Fig. 10:**
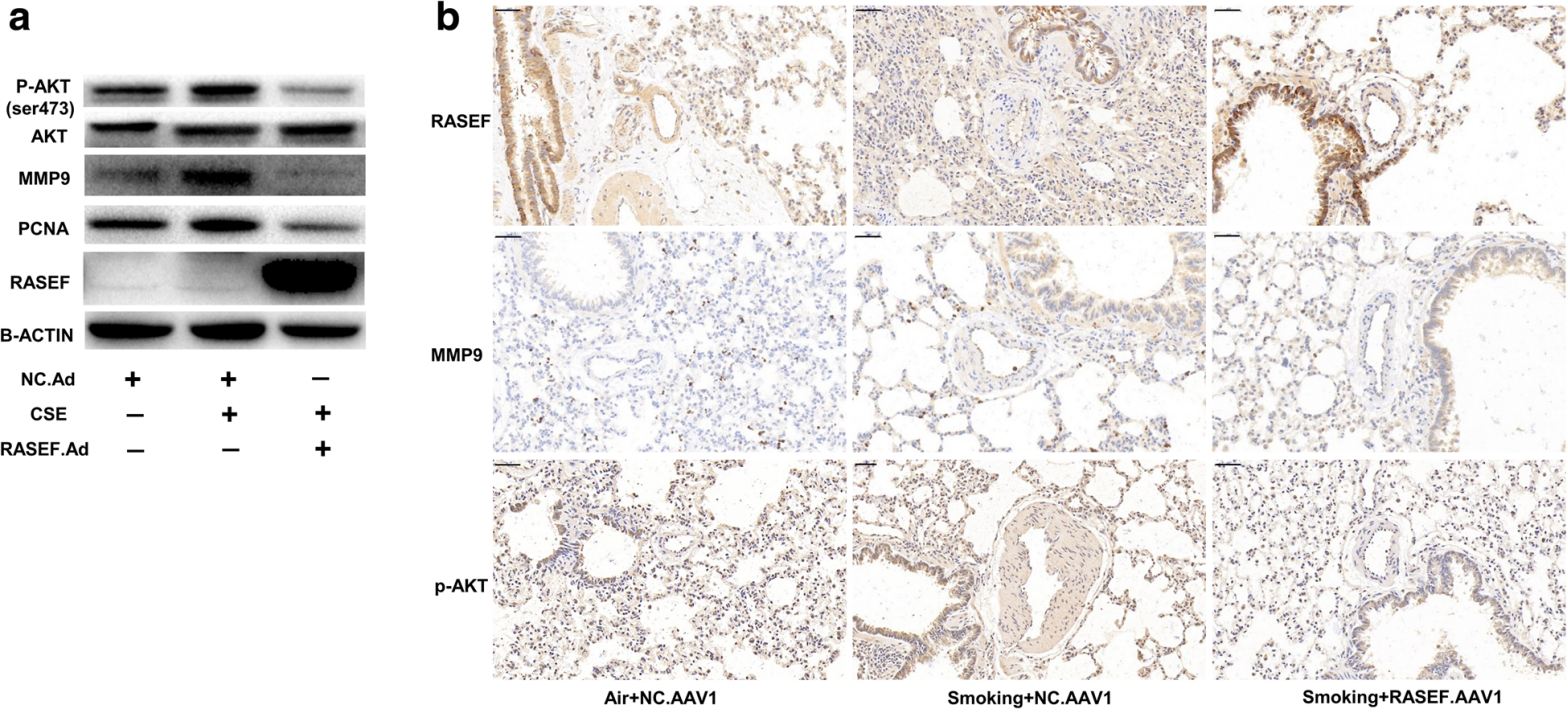
Effect of RASEF overexpression on cigarette smoke -induced AKT activation. **a** Transfection of HPASMCs with Ad.RASEF prevented 48-h CSE-induced increases in phospho-AKT (Ser473), PCNA, and MMP9 protein levels. **b** Protein expressions of RASEF, MMP9, and P-AKT (ser-473) in rat PAs were examined by immunochemistry. Scale bar = 50 μm

2% CSE increased PCNA and MMP9 protein levels in HPASMCs accompanied by upregulation of phospho-AKT (Ser473). However, these effects were inhibited by RASEF overexpression (Fig. 10a). And similar effects were also seen in vivo (Fig. 10b).

## Discussion

In this study**,** cigarette smoking (CS) exposure led to rat PH and induced proliferation and migration of HPASMCs, followed by increased DNMT1 mRNA and protein expression, *RASEF* silencing, and *RASEF* promotor hypermethylation in vivo and in vitro. However, pretreatment with either DNMT1 siRNA or 5-Aza inhibited CSE-induced HPASMCs proliferation, migration, and *RASEF* expression and methylation changes. Furthermore, RASEF overexpression improved CS-induced rat PH pathophysiology and inhibited CSE-induced HPASMCs proliferation and migration. Taken together, these results emphasize the important roles of RASEF methylation in CS-associated PH development.

The most commonly used rodent models of PH include those induced by monocrotaline or chronic hypoxia, which model Group 3 PH (PH due to lung diseases and/or hypoxia) [36, 37]. However, Group 3 PH also includes COPD-associated PH without hypoxemia, which requires greater consideration of the pathogenic roles of cigarette smoke [4]. Long-term (≥6 months) cigarette smoke exposure has been shown to cause emphysema in rodents [38], and short-term (3–4 months) exposure can induce rat PH [39, 40]. In the present study, rats exposed to CS for 3 months had increased RVSP, obvious PA remodeling, and RV hypertrophy.

As with other epigenetic changes (e.g., microRNA, histone modification, etc.), DNA methylation has also been implicated in the pathogenesis of PH, particularly in PA remodeling [19, 20]. Recent studies have shown that DNMT1 or its inhibitor, 5-Aza, influenced vascular smooth muscle phenotype and behaviors (e.g., proliferation and migration) resulting from hypoxia or platelet-derived growth factor treatment [21, 22]. The current results showed CS exposure increased DNMT1 mRNA and protein levels in rat PAs and HPASMCs, while DNMT1 knockdown inhibited CSE-induced proliferation and migration of HPASMCs. These results suggest that DNMT1 expression is closely related to CS-associated PA remodeling. However, the detailed mechanism of how DNA methylation influences CS-induced PA remodeling via regulation of DNMT1 expression remains unclear.

Although there are no reports regarding RASEF involvement in PA remodeling or PH pathology, other Rab family members have been found to be connected with vascular SMC function. For example, Rab5a knockdown has been shown to inhibit the proliferation and migration of human aorta SMCs, as well AKT activation [41], and Rab25 reportedly influences cerebral artery SMC vasoconstriction through regulating the abundance of Ca_v_1.2 [42]. Here, CS was found to decrease RASEF mRNA and protein expression in rat PAs and HPASMCs, while RASEF overexpression alleviated CS-induced PH in vivo and inhibited CSE-induced HPASMCs proliferation and migration in vitro. Hence, RASEF plays an important role in CS-associated PA remodeling.

Of note, *RASEF* silencing in melanoma resulted from its promotor hypermethylation, and was associated with the prognosis of melanoma patients [26, 43]. Interestingly, we found the methylation level of CpGs in RASEF promotor was significantly elevated in rat PAs in the smoking group. In order to illuminate the relationship between DNA methylation, DNMT1, and RASEF in CS-associated PA remodeling, we examined *RASEF* promoter methylation status and mRNA expression in HPASMCs under the combined stimuli including CSE and DNMT1siRNA or 5-aza. While CSE increased *RASEF* promotor methylation and RASEF expression in HPASMCs, pretreatment with either DNMT1 siRNA or 5-Aza inhibited these effects. Overall, these results suggest that CS induced PA remodeling likely via DNMT1-mediated *RASEF* hypermethylation in PASMCs.

**Fig. 11:**
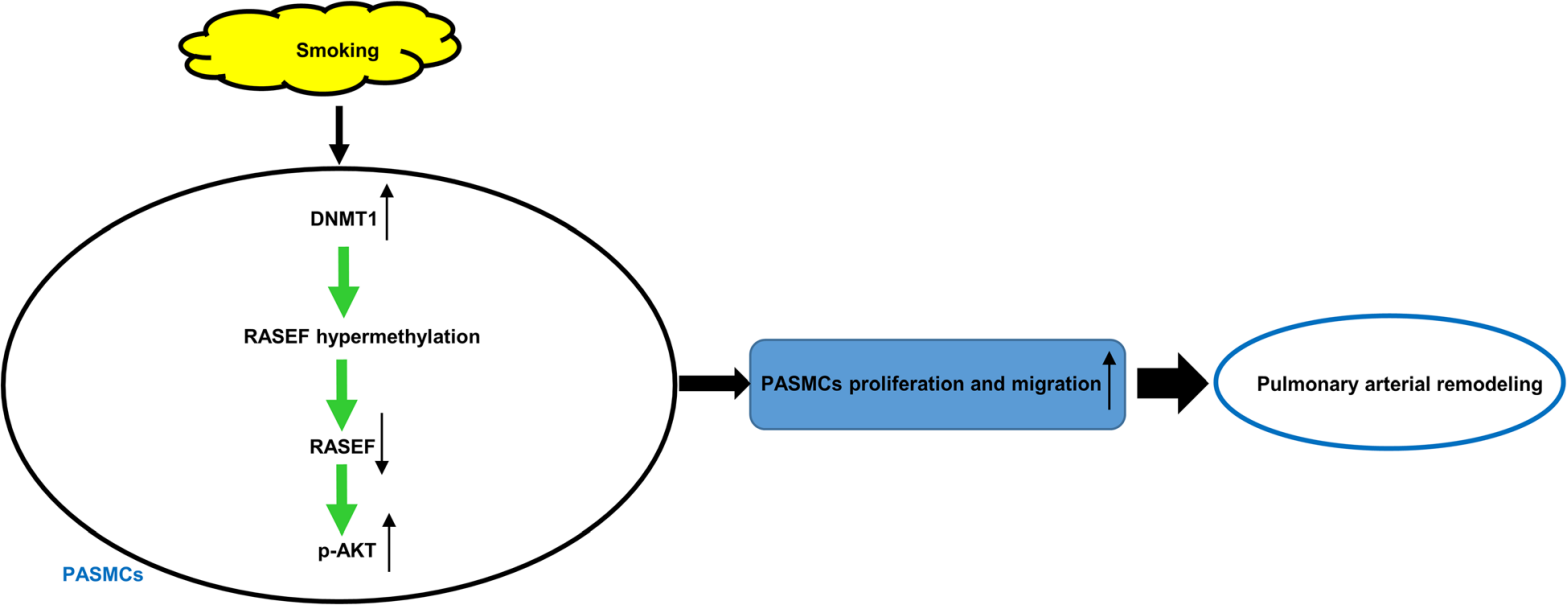
Schematic illustration of the potential role of DNA methylation of *RASEF* in the pathogenesis of pulmonary hypertension

AKT signaling plays an important role in vessel maturation [44] and vascular remodeling in PH models [45, 46]. Garat et al. demonstrated that tricirbine (an AKT inhibitor) attenuated hypoxia-induced PA media thickening and RV hypertrophy [47]. Although AKT has a vital role in vascular SMC proliferation [48] and migration [49], few studies have shown the link between AKT and CS-associated vascular remodeling [35]. In our study, CS was found to upregulate phospho-AKT (Ser 473), PCNA (proliferation marker), and MMP9 protein levels in HPASMCs, while RASEF overexpression inhibited these changes. Our previous study clarified the important role of MMP9 in PASMC migration in a monocrotaline-induced PH model [33], and some studies have reported AKT signaling could regulate MMP9 expression [50, 51]. The current result that CS upregulated MMP9 protein expression in HPASMCs is similar to that of Ghosh et al. [52]. More importantly, RASEF overexpression notably reversed CS-induced upregulation of P-AKT and MMP9 not only in vitro, but also in vivo. So we believe that RASEF plays a protective role in CS-induced PA remodeling possibly via regulation of P-AKT/MMP9 (Fig. 11).

There are also some limitations in our study. Though CSE treatment was found to enhance HPASMCs proliferation and migration as well as increase phospho-AKT (Ser 473), PCNA, and MMP9 levels, whether AKT directly or indirectly causes upregulation of MMP9 under PH conditions in vitro and/or in vivo to be seen. Nonetheless, Ghosh et al. previously reported AKT was the upstream of MMP9 in elastase-treated rat aortic SMCs [53]. In addition, whether RASEF, a member of Ras family, regulated AKT activation via Raf or other signal is uncertain and need to be studied in the future [54].

## Conclusions

DNMT1 mediated *RASEF* promotor hypermethylation plays an important role in cigarette smoke-induced PA remodeling. Therefore, RASEF may be a novel therapeutic target for treatment of cigarette smoke-associated PH.

## Additional file

Additional file 1: Methylation differences in CpG in PAs of the rats with different treatment (xls). The number 164, 165, 187 and 188 were from the air group, and the last (171,174, 189 and 197) were the smoking group rats. (XLS 1785 kb)

## Abbreviations

COPD: Chronic obstructive pulmonary disease
COPD-PH: pulmonary hypertension associated with COPD
CS: Cigarette smoking
CSE: Cigarette smoke extract
DNMT1: DNA methyltransferase 1
HPASMCs: Primary human pulmonary arterial smooth muscle cells
MCT: Monocrotaline
PAR: Pulmonary arterial remodeling
PAs: Pulmonary arteries
PASMR: Pulmonary arterial smooth muscle remodeling
PDGF: Platelet-derived growth factor
RASEF: RAS And EF-hand domain containing
RVHI: Right ventricle hypertrophy index
RVSP: Right ventricle systolic pressure
WT: Wall thickness
